# Living Alone and Alcohol-Related Mortality: A Population-Based Cohort Study from Finland

**DOI:** 10.1371/journal.pmed.1001094

**Published:** 2011-09-20

**Authors:** Kimmo Herttua, Pekka Martikainen, Jussi Vahtera, Mika Kivimäki

**Affiliations:** 1 Finnish Institute of Occupational Health, Helsinki, Finland; 2 Population Research Unit, Department of Social Research, University of Helsinki, Helsinki, Finland; 3 Department of Public Health, University of Turku and Turku University Hospital, Turku, Finland; 4 Department of Epidemiology and Public Health, University College London, London, United Kingdom; 5 Department of Behavioral Sciences, University of Helsinki, Helsinki, Finland; University of Cambridge, United Kingdom

## Abstract

**Background:**

Social isolation and living alone are increasingly common in industrialised countries. However, few studies have investigated the potential public health implications of this trend. We estimated the relative risk of death from alcohol-related causes among individuals living alone and determined whether this risk changed after a large reduction in alcohol prices.

**Methods and Findings:**

We conducted a population-based natural experimental study of a change in the price of alcohol that occurred because of new laws enacted in Finland in January and March of 2004, utilising national registers. The data are based on an 11% sample of the Finnish population aged 15–79 y supplemented with an oversample of deaths. The oversample covered 80% of all deaths during the periods January 1, 2000–December 31, 2003 (the four years immediately before the price reduction of alcohol), and January 1, 2004–December 31, 2007 (the four years immediately after the price reduction). Alcohol-related mortality was defined using both underlying and contributory causes of death. During the 8-y follow-up about 18,200 persons died due to alcohol-related causes. Among married or cohabiting people the increase in alcohol-related mortality was small or non-existing between the periods 2000–2003 and 2004–2007, whereas for those living alone, this increase was substantial, especially in men and women aged 50–69 y. For liver disease in men, the most common fatal alcohol-related disease, the age-adjusted risk ratio associated with living alone was 3.7 (95% confidence interval 3.3, 4.1) before and 4.9 (95% CI 4.4, 5.4) after the price reduction (*p*<0.001 for difference in risk ratios). In women, the corresponding risk ratios were 1.7 (95% CI 1.4, 2.1) and 2.4 (95% CI 2.0, 2.9), respectively (*p* ≤ 0.01). Living alone was also associated with other mortality from alcohol-related diseases (range of risk ratios 2.3 to 8.0) as well as deaths from accidents and violence with alcohol as a contributing cause (risk ratios between 2.1 and 4.7), both before and after the price reduction.

**Conclusions:**

Living alone is associated with a substantially increased risk of alcohol-related mortality, irrespective of gender, socioeconomic status, or the specific cause of death. The greater availability of alcohol in Finland after legislation-instituted price reductions in the first three months of 2004 increased in particular the relative excess in fatal liver disease among individuals living alone.

*Please see later in the article for the Editors' Summary*

## Abstract 2

Kimmo Herttua and colleagues showed that living alone is associated with a substantially increased risk of alcohol-related mortality, irrespective of gender, socioeconomic status, or cause of death, and that this effect was exacerbated after a price reduction in alcohol in 2004.

## Editors' Summary

**Background:**

Throughout most of human history, people have lived in tight-knit communities where there was likely to be someone to turn to for help, advice, or company. But the modern way of life in industrialized countries is greatly reducing the quantity and quality of social relationships. Instead of living in extended families, many people now live miles away from their relatives, often living and working alone. Others commute long distances to work, which leaves little time for socializing with friends or relatives. And many delay or forgo getting married and having children. Consequently, loneliness and social isolation are getting more common. In the UK, according to a recent survey by the Mental Health Foundation, 10% of people often feel lonely, a third have a close friend or relative who they think is very lonely, and half think people are getting lonelier in general. Similarly, over the past two decades, there has been a three-fold increase in the number of Americans who say they have no close confidants.

**Why Was This Study Done?:**

Some experts think that loneliness is bad for human health. They point to studies that show that people with fewer social relationships die earlier on average than people with more social relationships. But does loneliness increase the risk of dying from specific causes? It is important to investigate the relationship between loneliness and cause-specific mortality (death) because, if for example, loneliness increases the risk of dying from alcohol-related causes (heavy drinking causes liver and heart damage, increases the risk of some cancers, contributes to depression, and increases the risk of death by violence or accident), doctors could advise their patients who live alone about safe drinking. But, although loneliness is recognized as both a contributor to and a consequence of alcohol abuse, there have been no large, population-based studies on the association between living alone and alcohol-related mortality. In this population-based study, the researchers estimate the association between living alone (an indicator of a lack of social relationships) and death from alcohol-related causes in Finland for four years before and four years after an alcohol price reduction in 2004 that increased alcohol consumption.

**What Did the Researchers Do and Find?:**

The researchers obtained information on about 80% of all people who died in Finland between 2000 and 2007 from Statistics Finland, which collects official Finnish statistics. During this period, about 18,200 people (two-thirds of whom lived alone) died from underlying alcohol-related causes (for example, liver disease and alcoholic poisoning) or contributory alcohol-related causes (for example, accidents, violence, and cardiovascular disease, with alcohol as a contributing cause). Among married and cohabiting people, the rate of alcohol-related mortality was similar in 2000–2003 and 2004–2007 but for people living alone (particularly those aged 50–69 years) the 2004 alcohol price reduction substantially increased the alcohol-related mortality rate. For liver disease in men, the risk ratio associated with living alone was 3.7 before and 4.9 after the price reduction. That is, between 2000 and 2003, men living alone were 3.7 times more likely to die of liver disease than married or cohabiting men; between 2004 and 2007, they were 4.9 times more likely to die of liver disease. In women, the corresponding risk ratios for liver disease were 1.7 and 2.4, respectively. Living alone was also associated with an increased risk of dying from other alcohol-related diseases and accidents and violence both before and after the price reduction.

**What Do These Findings Mean?:**

These findings indicate that, in Finland, living alone is associated with an increased risk of alcohol-related mortality. Because of the study design, it is impossible to say whether living alone is a cause or a consequence of alcohol abuse, but the greater increase in alcohol-related deaths (particularly fatal liver disease) among people living alone compared to married and cohabiting people after the alcohol price reduction suggests that people living alone are more vulnerable to the adverse effects of increased alcohol availability. Further research in other countries is now needed to identify whether living alone is a cause or effect of alcohol abuse and to extend these findings to cultures where the pattern of alcohol consumption is different. However, the findings of this natural experiment suggest that living alone should be regarded as a potential risk marker for death from alcohol-related causes.

**Additional Information:**

Please access these websites via the online version of this summary at http://dx.doi.org/10.1371/journal.pmed.1001094.

- The Mental Health America Live Your Life Well webpage includes information about how social relationships improve mental and physical health
- The Mental Health Foundation (a UK charity) presents the report The Lonely Society?
- The US National Institute on Alcohol Abuse and Alcoholism has information about alcohol and its effects on health
- The US Centers for Disease Control and Prevention has a website on alcohol and public health that includes information on the health risks of excessive drinking
- The UK National Health Service Choices website provides detailed information about drinking and alcohol, including information on the risks of drinking too much, and personal stories about alcohol problems, including stories from people living alone (My drinks diary shock and I used to drink all day)
- MedlinePlus provides links to many other resources on alcohol

## Introduction

The modern way of life in industrialised countries is greatly reducing the quantity and quality of social relationships [1]. Fewer people live in extended families, and many delay and altogether avoid getting married and having children [2],[3]. There is reason to believe that people are becoming more socially isolated [1]. Over the past two decades in the US, for example, there has been a 3-fold increase in the number of Americans who say they have no close confidants [2]. In the UK, according to a recent survey by the Mental Health Foundation, 10% of people often feel lonely, a third have a close friend or relative who they think is very lonely, and half think that people are getting lonelier in general [4].

A number of studies suggest that living alone is a risk factor for mortality, with the exception of aged population [5]–[7]. However, research on cause-specific mortality is scarce, and although loneliness has for a long time been recognised as a contributing or maintaining factor in alcohol abuse as well as a consequence of alcohol abuse [8], we are not aware of large-scale population-based studies on the association between living alone and alcohol-related mortality. Such an association is highly plausible given the link between living alone and depression [9] and the possibility that excessive alcohol use may operate as self-medication in lonely and depressive individuals [8].

In this population-based study of Finnish residents we therefore aimed to estimate the association between living alone and mortality from different alcohol-related causes of death. We also took advantage of the substantial price reduction in alcohol prices in Finland during the study period to determine whether increased availability of alcohol further increases the risk of alcohol-related death among individuals living alone.

## Methods

### Ethics Statement

Statistics Finland gave ethical approval for the study.

### Study Context and Study Population

The changes in Finnish alcohol legislation that occurred in 2004 can be considered as a natural experiment. On January 1, 2004, it became legal to import practically unlimited amounts of alcoholic beverages for one's own use from other EU countries without paying further taxes. A second law implemented on March 1, 2004, reduced taxes on alcohol by an average of 33%: the off-premise retail price (i.e., the price of alcoholic beverages that will be consumed away from the site of sale, e.g., those sold in state monopoly stores or supermarkets) of spirits went down by 28%–36%, wines by 3%, beer by 13%, and other alcoholic beverages by 7%–28% [10]. The reason for the tax cuts was that Estonia, a neighbouring country of Finland, joined the EU on May 1, which was expected to substantially affect the Finnish alcohol market because of the proximity of the two countries and the considerably lower price of alcohol in Estonia. The total per-capita alcohol consumption (recorded and unrecorded) in Finland is estimated to have increased approximately 10% in 2004 to over 10 l per capita, and has remained more or less on that level since then [11].

All the data for this study were obtained from the Statistics Finland Labour Market data file covering all Finns with a linkage to death records in the period January 1, 2000–December 31, 2007. The linkage was carried out by Statistics Finland by means of personal identification codes (permission TK 53-508-09). Owing to data-protection regulations concerning living individuals, Statistics Finland provided only an 11% sample of the whole dataset. In order to maintain power in the mortality analyses, we further obtained an oversample of those who died in the period January 1, 2000–December 31,2007—for whom the data-protection regulations are less strict—and thus covered altogether 80% of all deaths in that period. We used sampling weights, constructed from the sampling probabilities, in order to take account of the sampling design. Thus, the results derived from the analyses of this study are nationally representative. We restricted the sample in this study to individuals aged 15–79 y. The baseline consisted of all individuals of that age on December 31, 1999. Individuals reaching the age of 15 y during the study entered the analysis on the first day of the month of their birthday, and those reaching the age of 80 y became censored on the first day of the month in which they turned 80 y.

### Follow-Up for Alcohol-Related Mortality

Causes of death were classified according to the Finnish edition (FCD) of the *International Statistical Classification of Diseases and Related Health Problems, 10th revision* (ICD-10). Alcohol-related deaths were defined as those for which there was a reference to alcohol on the death certificate as the underlying or one of the contributory causes of death. Estimating alcohol-related mortality on the basis of both the underlying and contributory causes yields more versatile and comprehensive data than the standard method based solely on the underlying cause, particularly in Finland, where death certificates record alcohol intoxication as a contributory cause more frequently and accurately than in most other countries [12],[13]. Frequent use of medicolegal autopsy is one of the major factors enabling the proper attribution of alcohol intoxication as a contributory cause of death. Medicolegal autopsies were carried out in 91% of all cases of accidental or violent death occurring among people aged under 65 y in 2007 [14], and in more than 60% of all deaths in 1987–2003 [15]. The high quality of the cause-specific death register in Finland was demonstrated in international comparisons of death registration [16]. Finland was ranked among the best countries regardless of the indicator of data quality used [16].

The total pool of alcohol-related deaths used here consists of the following two main categories: (1) the underlying cause of death was an alcohol-attributable disease or fatal alcohol poisoning (ICD-10 code X45); and (2) the underlying cause was not alcohol related, but a contributory cause was an alcohol-attributable disease or alcohol intoxication (ICD-10 code F100). The first group constituted 46% of all alcohol-related deaths (*n* = 18,246). Of all deaths in which the underlying cause was alcohol attributable, 48% referred to alcoholic liver disease (ICD-10 code K70), 38% to fatal alcohol poisoning (ICD-10 code X45) or to alcohol dependence syndrome (ICD-10 code F102), 5% to alcoholic cardiomyopathy (ICD-10 code I426), 5% to alcoholic diseases of the pancreas (ICD-10 codes K852 and K860), 2% to other mental and behavioural disorders due to alcohol (ICD-10 codes F101 and F103–F109), and 2% to a few rarely occurring categories (ICD-10 codes K292, G312, G4051, G621, and G721). In the second group, the underlying cause was accident or violence in 50% and cardiovascular disease in 34% of the cases. Cardiovascular diseases consist of the following categories: ischemic heart diseases (ICD-10 codes I20–I25), other heart diseases excluding rheumatic heart diseases (ICD-10 codes I30–I425 and I427–I52), cerebrovascular diseases (ICD-10 codes I60–I69), and other diseases of the circulatory system (ICD-10 codes I00–I15, I26–I28, and I70–I99).

We used alcoholic liver diseases instead of all liver diseases because there seems not to be any strong tendency to underreport alcoholic cases in Finland: for example, in 2006, 98% of deaths due to liver cirrhosis among men aged <65 y were classified as alcohol related on the death certificate [17]. The proportion of all alcohol-related deaths among men was 83%.

### Assessment of Living Arrangements and Demographic Characteristics

Data on living arrangements and demographic characteristics were registered at the turn of each year. To allow changes in the measures during the follow-up, all the variables were included in the analyses as time-varying covariates. Living arrangements were classified into two groups: (1) married or cohabiting individuals and (2) persons living alone. Statistics Finland defined cohabiters as persons living in the same dwelling, aged 18 y or over, of different sex, not being siblings, and with an age difference that does not exceed 15 y (additional people could also live in the home). People not belonging to these two categories were excluded from the analyses due to heterogeneity of this group (altogether 21% of all persons). Demographic factors included sex, age group (5-y categories), and socioeconomic characteristics, and were treated as covariates in the analysis. The four educational categories were based on the highest level of education achieved, obtained from the National Register of Completed Education and Degrees: basic education, secondary education, lower tertiary education, and higher tertiary education (the equivalent of graduate school in the US educational system). Occupational social class was divided into six categories: upper white-collar, lower white-collar, skilled worker, unskilled worker, self-employed, and other. Economically inactive individuals were categorised according to occupation held at the time of a previous measurement point or according to the head of household. Income was measured as individual taxable income, comprising all forms of taxable income, including wages, capital income, and taxable income transfers, and excluding certain social benefits and allowances not subject to taxation. In the analyses we used income deciles with cut-off points calculated from the combined data for men and women for each year separately.

### Statistical Methods

All the analyses were conducted separately for men and women, using Stata, version 10 (Stata Corporation). We calculated hazard ratios with 95% confidence intervals (CIs) from Cox regression models to assess the relative differences in alcohol-related mortality outcomes between those living alone and married or cohabiting persons. We adjusted these models for age, education, social class, and income. Five-year age groups were treated as continuous variables in the Cox models. We also performed analyses by using sex, continuous age term, and squared age term as covariates (Tables S6–S9). However, the estimates were little changed. Calendar time was used as the time scale in the analyses. In order to determine the relative effect of the alcohol price reduction on alcohol-related mortality according to living alone versus married or cohabiting, we included calendar period/living alone interaction terms in the models, and used likelihood ratio tests to derive the *p-*values.

## Results

### Descriptive Results

The total number of alcohol-related deaths in the sample of married or cohabiting persons and the sample of those living alone was 6,731 and 11,515, respectively, among individuals aged 15–79 y in 2000–2007 (Tables 1 and 2). Of these deaths, 77% and 85% occurred in men. The number of deaths and death rate per 100,000 person-years were highest among 50- to 69-y-olds, with the exception of women living alone aged 40–49 y, whose mortality rate was level with that of 50- to 59-y-olds. Compared to cohabiters, crude death rates among individuals living alone were about 5-fold higher for men and 3-fold higher for women.

**Table 1:** Distribution of sample population, number of alcohol-related deaths, and mortality rates per 100,000 person-years in 2000–2007 according to age group, education, social class, and income, for men aged 15–79 y living alone or married or cohabiting.

Variable	Subcategory	Married or Cohabiting			Living Alone		
		Percent	Deaths^a^	Mortality Rate	Percent	Deaths^a^	Mortality Rate
**Age group, years**	15–39	30.2	469	23.2	40.9	1,037	105.3
	40–49	22.2	911	61.4	19.1	2,142	466.7
	50–59	23.1	1,882	122.3	19.2	3,550	774.9
	60–69	15.2	1,373	136.6	12.1	2,199	762.4
	70–79	9.3	562	92.2	8.7	842	412.8
**Education**	Upper tertiary	9.2	233	38.1	5.6	201	148.7
	Lower tertiary	20.1	657	49.1	13.8	915	277.2
	Secondary	38.7	1,764	68.3	45.0	3,650	337.6
	Basic	32.2	2,543	119.7	35.6	5,004	590.5
**Social class**	Upper white-collar	18.4	561	45.6	11.3	614	226.1
	Lower white-collar	19.8	847	64.4	16.2	1,204	309.4
	Skilled worker	24.6	1,607	98.4	24.3	3,071	527.9
	Unskilled worker	17.0	1,183	104.4	25.3	3,375	558.1
	Self-employed	15.4	806	78.7	10.1	876	362.1
	Other	4.8	193	59.9	12.7	630	206.7
**Income**	1st decile (highest)	22.6	498	33.1	10.8	296	113.6
	2nd decile	17.8	468	39.4	12.0	364	126.0
	3rd decile	13.6	440	48.6	10.8	425	164.0
	4th decile	10.6	490	69.2	9.4	513	226.7
	5th decile	10.3	659	96.5	10.0	862	360.0
	6th decile	9.0	795	132.6	10.8	1,213	473.0
	7th decile	6.8	672	149.3	10.4	1,463	592.1
	8th decile	4.7	668	213.6	12.0	2,440	855.3
	9th decile	3.0	372	185.9	10.0	1,789	747.3
	10th decile	1.6	135	129.0	3.8	405	443.1
**Total**		100	5,197	78.1	100	9,770	408.1

a Numbers of deaths are those observed in the original sample, whereas other columns are based on analyses that use weights to account for the different sampling probabilities (see Methods).

**Table 2:** Distribution of sample population, number of alcohol-related deaths, and mortality rates per 100,000 person-years in 2000–2007 according to age group, education, social class, and income, for women aged 15–79 y living alone or married or cohabiting.

Variable	Subcategory	Married or Cohabiting			Living Alone		
		Percent	Deaths^a^	Mortality Rate	Percent	Deaths^a^	Mortality Rate
**Age group, years**	15–39	34.5	127	5.4	26.9	133	17.8
	40–49	22.5	329	21.5	10.6	349	118.7
	50–59	21.9	594	40.0	18.6	626	121.1
	60–69	13.6	369	40.0	19.4	435	80.8
	70–79	7.4	115	23.0	24.5	202	30.0
**Education**	Upper tertiary	7.9	38	7.1	6.1	34	20.1
	Lower tertiary	25.2	196	11.5	18.0	173	34.6
	Secondary	38.0	476	18.5	34.6	556	57.8
	Basic	28.9	824	42.1	41.3	982	86.0
**Social class**	Upper white-collar	14.8	118	11.8	11.9	110	33.3
	Lower white-collar	40.5	549	20.0	36.8	593	58.0
	Skilled worker	8.2	183	32.8	9.6	229	86.1
	Unskilled worker	18.3	452	36.5	22.7	580	92.4
	Self-employed	10.3	155	22.3	7.9	115	52.4
	Other	7.9	77	14.4	11.1	118	38.4
**Income**	1st decile (highest)	6.5	27	6.2	5.4	33	21.9
	2nd decile	9.1	63	10.2	7.9	59	27.0
	3rd decile	12.4	80	9.5	9.8	75	27.7
	4th decile	14.7	99	9.9	11	83	27.2
	5th decile	13.3	114	12.6	12.5	166	48.0
	6th decile	11.7	221	27.9	14.9	219	53.0
	7th decile	11.7	282	35.7	15.8	296	67.6
	8th decile	10.7	324	44.9	12.5	394	114.4
	9th decile	7.4	238	47.4	7.8	347	160.3
	10th decile	2.6	86	48.8	2.5	73	106.6
**Total**		100	1,534	22.6	100	1,745	63.0

a Numbers of deaths are those observed in the original sample, whereas other columns are based on analyses that use weights to account for the different sampling probabilities (see Methods).

There was a graded association between education and alcohol-related mortality among both married or cohabiting and alone-living men and women (Tables 1 and 2). The rate of alcohol-related mortality among persons with basic education was three to six times greater compared to that in the highest education group. Within educational groups, mortality rates among persons living alone were 2- to 6-fold higher than among married or cohabiting individuals. The pattern of associations was quite similar with regard to social class: the rate of alcohol-related mortality among unskilled workers was 2- to 3-fold greater than among upper white-collar workers, whereas the rate ratios within social-class groups varied from 2.3 to 5.4. These rate ratios were generally higher among men than women. With regard to personal income, the alcohol-related mortality rates of the three lowest income deciles were approximately 7- to 8-fold greater than those of the highest decile, whereas the rate ratios within income deciles varied from 1.9 to 4.0. These findings suggest that socioeconomic factors are associated with alcohol-related mortality irrespective of living arrangements.

### Association between Living Alone and Alcohol-Related Mortality before and after Alcohol Price Change

**Figure 1:**
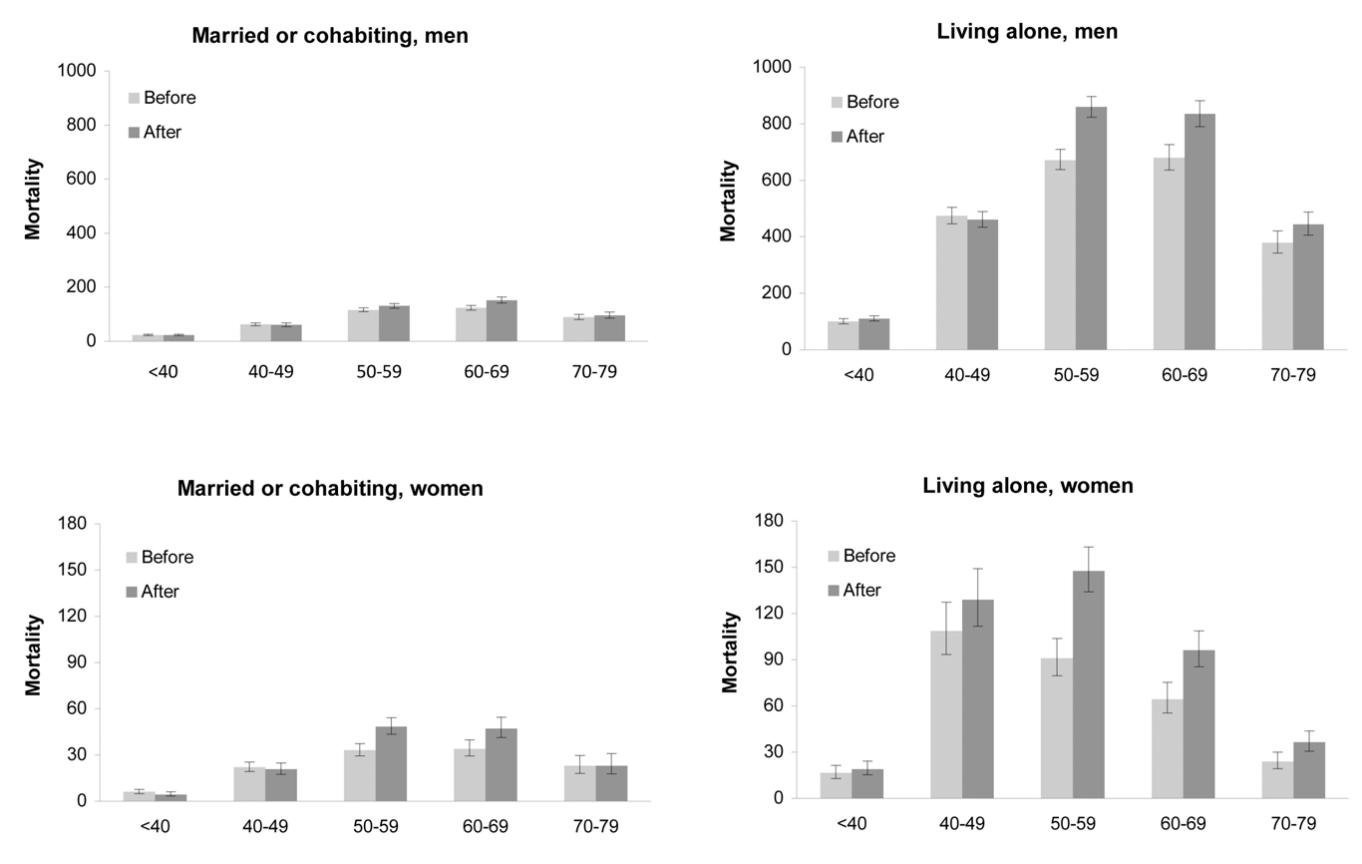
Number of alcohol-related deaths per 100,000 person-years among persons aged 15–79 y before (2000–2003) and after (2004–2007) the reduction in alcohol prices. *x*-Axes give age groups, in years. Bars indicate 95% CIs. (A) Mortality among men living with a spouse (number of deaths: *n = *5,197); (B) mortality among men living alone (*n = *9,770); (C) mortality among women living with a spouse (*n = *1,534); (D) mortality among women living alone (*n = *1,745).

The change in total alcohol-related mortality from the time period 2000–2003 to the time period 2004–2007 varied considerably by age (Figure 1). Among married or cohabiting men and women aged under 50 y or over 70 y, the increase in mortality per 100,000 was ≤7, whereas the corresponding figure varied between 13 and 28 among the 50- to 69-y-olds. Among persons living alone, mortality rate increased more (32–187 deaths per 100,000) in men and women aged 50–69 y. These findings suggest that the increase in alcohol-related mortality in relation to price reduction was dependent on living arrangements.

As shown in Table 3, the risk of alcohol-related mortality in men was substantially higher for those living alone compared with married or cohabiting men. Before the reduction in alcohol prices, the strength of the association between living alone and alcohol-related deaths varied depending on the cause of death. Age-adjusted risk ratios were highest for alcohol dependence and poisoning and other alcohol-related diseases, 7.17 (95% CI 6.35, 8.10) and 7.32 (95% CI 6.06, 8.84), respectively, and lowest for liver disease, 3.70 (95% CI 3.31, 4.14). Further adjustments for education and social class did not largely affect the association between living alone and alcohol-related mortality, whereas controlling for income attenuated this association: risk ratios for mortality from alcohol dependence and poisoning, other alcohol-related diseases, and liver disease were reduced to 5.17 (95% CI 4.57, 5.84), 5.62 (95% CI 4.63, 6.84), and 2.85 (95% CI 2.54, 3.19), respectively.

**Table 3:** Relative alcohol-related mortality for living alone versus married or cohabiting in men aged 15–79 y before (2000–2003) and after (2004–2007) the alcohol price reduction.

Time Period	Cause of Death	Deaths^a^	Mortality Rate^b^	RRs for Living Alone versus Married or Cohabiting					
				Model 1		Model 2		Model 3	
				RR	95% CI	RR	95% CI	RR	95% CI
**Before price reduction**	**Total alcohol-related mortality**								
	Married or cohabiting	2,804	72.4	1.00		1.00		1.00	
	Living alone	4,199	418.3	5.44	5.18–5.72	5.18	4.93–5.44	4.15	3.95–4.36
	**Liver disease**								
	Married or cohabiting	627	16.1	1.00		1.00		1.00	
	Living alone	632	64.8	3.70	3.31–4.14	3.57	3.20–4.00	2.85	2.54–3.19
	**Dependence and poisoning**								
	Married or cohabiting	393	10.0	1.00		1.00		1.00	
	Living alone	788	78.5	7.17	6.35–8.10	6.85	6.06–7.74	5.17	4.57–5.84
	**Other alcohol-related diseases**								
	Married or cohabiting	162	4.1	1.00		1.00		1.00	
	Living alone	325	33.1	7.32	6.06–8.84	7.00	5.78–8.46	5.62	4.63–6.84
	**Cardiovascular diseases**								
	Married or cohabiting	513	13.3	1.00		1.00		1.00	
	Living alone	854	87.8	6.21	5.56–6.93	5.95	5.32–6.65	5.00	4.47–5.60
	**Accidents and violence**								
	Married or cohabiting	901	23.6	1.00		1.00		1.00	
	Living alone	1,200	113.8	4.63	4.24–5.05	4.33	3.96–4.73	3.58	3.28–3.92
**After price reduction**	**Total alcohol-related mortality**								
	Married or cohabiting	2,393	79.4	1.00		1.00		1.00	
	Living alone	5,571	489.6	5.86	5.57–6.17	5.54	5.26–5.83	4.28	4.06–4.52
	*p-*value^c^				0.051		0.073		0.408
	**Liver disease**								
	Married or cohabiting	611	20.0	1.00		1.00		1.00	
	Living alone	1,160	103.6	4.85	4.37–5.38	4.66	4.20–5.17	3.52	3.16–3.91
	*p-*value^c^				<0.001		0.001		0.003
	**Dependence and poisoning**								
	Married or cohabiting	353	11.8	1.00		1.00		1.00	
	Living alone	956	85.1	6.77	5.96–7.69	6.35	5.58–7.23	4.61	4.04–5.26
	*p-*value^c^				0.465		0.426		0.197
	**Other alcohol-related diseases**								
	Married or cohabiting	136	4.5	1.00		1.00		1.00	
	Living alone	440	39.2	7.97	6.52–9.74	7.54	6.16–9.22	5.83	4.75–7.15
	*p-*value^c^				0.544		0.568		0.744
	**Cardiovascular diseases**								
	Married or cohabiting	428	13.9	1.00		1.00		1.00	
	Living alone	1,141	101.4	7.00	6.23–7.86	6.75	6.00–7.59	5.59	4.97–6.30
	*p-*value^c^				0.144		0.157		0.274
	**Accidents and violence**								
	Married or cohabiting	703	24.2	1.00		1.00		1.00	
	Living alone	1,381	117.2	4.70	4.27–5.18	4.34	3.94–4.79	3.44	3.12–3.80
	*p-*value^c^				0.989		0.907		0.565

Model 1: adjusted for age. Model 2: adjusted for age, education, and social class. Model 3: adjusted for age, education, social class, and income.

a Numbers of deaths are those observed in the original sample.

b Mortality rates (deaths per 100,000) are adjusted for age.

c *p-*Value for change in difference in excess mortality for those living alone compared to married or cohabiting persons.

RR, risk ratio.

After the alcohol price reduction, age-adjusted risk ratios for all specific causes were mainly slightly higher than before the price reduction, but interaction models revealed that the increase in the risk ratio was statistically significant only for mortality due to liver disease, even after controlling for age, education, social class, and income.

Table 4 shows the substantial association of living alone with all alcohol-related mortality categories among women, although these associations were generally not as strong as among men. Age-adjusted risk ratio was highest in alcohol dependence and poisoning, 2.54 (95% CI 2.02, 3.20), and lowest in liver disease, 1.69 (95% CI 1.36, 2.09), before the price reduction. Further adjustments with socioeconomic factors did not remove these associations.

**Table 4:** Relative alcohol-related mortality for living alone versus married and cohabiting in women aged 15–79 y before (2000–2003) and after (2004–2007) the price reduction.

Time Period	Cause of Death	Deaths^a^	Mortality Rate^b^	RRs for Living Alone versus Married or Cohabiting					
				Model 1		Model 2		Model 3	
				RR	95% CI	RR	95% CI	RR	95% CI
**Before price reduction**	**Total alcohol-related mortality**								
	Married or cohabiting	791	20.9	1.00		1.00		1.00	
	Living alone	691	60.1	2.18	1.95–2.43	2.11	1.89–2.35	2.17	1.95–2.42
	**Liver disease**								
	Married or cohabiting	248	6.7	1.00		1.00		1.00	
	Living alone	177	16.0	1.69	1.36–2.09	1.66	1.34–2.04	1.77	1.44–2.17
	**Dependence and poisoning**								
	Married or cohabiting	169	4.3	1.00		1.00		1.00	
	Living alone	162	15.2	2.54	2.02–3.20	2.46	1.96–3.10	2.52	2.01–3.16
	**Other alcohol-related diseases**								
	Married or cohabiting	34	0.9	1.00		1.00		1.00	
	Living alone	31	3.1	2.29	1.35–3.89	2.18	1.29–3.66	2.25	1.35–3.74
	**Cardiovascular diseases**								
	Married or cohabiting	87	2.3	1.00		1.00		1.00	
	Living alone	104	7.2	2.33	1.71–3.19	2.28	1.68–3.11	2.42	1.78–3.27
	**Accidents and violence**								
	Married or cohabiting	196	4.9	1.00		1.00		1.00	
	Living alone	147	13.3	2.10	1.68–2.63	2.02	1.62–2.53	2.03	1.62–2.53
**After price reduction**	**Total alcohol-related mortality**								
	Married or cohabiting	743	25.6	1.00		1.00		1.00	
	Living alone	1,054	83.8	2.56	2.31–2.85	2.44	2.19–2.70	2.47	2.23–2.74
	*p-*value^c^				0.013		0.027		0.063
	**Liver disease**								
	Married or cohabiting	251	8.6	1.00		1.00		1.00	
	Living alone	328	26.2	2.38	1.98–2.86	2.27	1.89–2.72	2.32	1.93–2.77
	*p-*value^c^				0.013		0.020		0.036
	**Dependence and poisoning**								
	Married or cohabiting	141	4.7	1.00		1.00		1.00	
	Living alone	188	16.5	2.52	1.97–3.22	2.38	1.86–3.05	2.41	1.89–3.08
	*p-*value^c^				0.859		0.944		0.910
	**Other alcohol-related diseases**								
	Married or cohabiting	26	0.9	1.00		1.00		1.00	
	Living alone	60	5.0	4.21	2.54–6.98	3.96	2.38–6.60	4.05	2.43–6.72
	*p-*value^c^				0.076		0.085		0.102
	**Cardiovascular diseases**								
	Married or cohabiting	91	3.3	1.00		1.00		1.00	
	Living alone	158	10.4	2.69	2.01–3.59	2.58	1.94–3.44	2.68	2.03–3.56
	*p-*value^c^				0.569		0.650		0.710
	**Accidents and violence**								
	Married or cohabiting	169	5.9	1.00		1.00		1.00	
	Living alone	222	19.0	2.58	2.07–3.22	2.42	1.94–3.01	2.39	1.92–2.98
	*p-*value^c^				0.157		0.194		0.242

Model 1: adjusted for age. Model 2: adjusted for age, education, and social class. Model 3: adjusted for age, education, social class, and income.

a Numbers of deaths are those observed in the original sample.

b Mortality rates (deaths per 100,000) are adjusted for age.

c *p-*Value for change in difference in excess mortality for those living alone compared to married or cohabiting persons.

RR, risk ratio.

After the price reduction, age-adjusted risk ratios were highest for other alcohol-related diseases, 4.21 (95% CI 2.54, 6.98), and lowest for liver disease, 2.38 (95% CI 1.98, 2.86). Control for socioeconomic factors did not largely attenuate these ratios. As in men, the relationship between living alone and alcohol-related mortality from liver cirrhosis was strengthened after the price reduction.

### Sensitivity Analysis

Coding artefacts in the death certificates are a potential source of type I error (false positive) if certifying doctors were more likely to ascribe death to an “alcohol related” cause in persons known to be living alone. To examine this possibility, we performed a sensitivity analysis using more inclusive death categories (i.e., mortality from gastro-intestinal causes, neuro-psychiatric causes, intentional injuries, non-intentional injuries, and non-specific causes), with and without alcohol-related deaths included in each category. If the certifying doctors were biased towards ascribing deaths to “alcohol related” causes in persons living alone, then (1) mortality associated with a broader death category that includes alcohol-related deaths as a subset should be similar among alone-living participants and those married or cohabiting, because ascribing bias does not increase the total number of cases in the broader death category; and (2) mortality associated with a broader category, when excluding alcohol-related deaths, should be lower among those living alone, given that the ascribing bias disproportionally inflated the number of alcohol-related deaths in this group. As shown in Tables S1–S5, neither of these predictions were true. First, living alone was associated with an increased mortality risk when alcohol-related deaths were included as a subcategory (Tables S1–S3). Second, when alcohol-related deaths were excluded from the broader death categories, the relative risk was not lower among persons living alone compared with those married or cohabiting (Tables S4 and S5). The only exception to this general pattern was mortality from neuro-psychiatric causes, which appeared to be slightly lower among participants living alone, both before and after including alcohol-related deaths in the category. However, of the 1,344 and 1,049 neuro-psychiatric deaths in men and women before the price reduction, only 31 (in men) and seven (in women) were due to alcohol-related causes. After the price reduction, 1,236 and 923 neuro-psychiatric deaths were recorded in men and women; only 37 and 11 were from alcohol-related causes. Any bias related to such a small proportion of alcohol-related deaths is unlikely to explain the lower neuro-psychiatric death rates among men and women living alone.

## Discussion

### Principal Findings of Study

In this population-based natural experimental study from Finland we sought to estimate the relative risk of death from alcohol-related causes among persons living alone versus cohabiting, and the change in this risk after a substantial reduction in alcohol prices. We found a marked increase in alcohol-related mortality after the price reduction for those living alone and aged 50–69 y but not for married or cohabiting persons (all ages). For liver disease, which is the most common fatal alcohol-related disease, the age-adjusted risk ratio associated with living alone versus being married or cohabiting was 3.7 before and 4.9 after the reduction in alcohol prices among men. The corresponding relative risks were 1.7 and 2.4 among women. Living alone was also associated with deaths from other alcohol-related diseases, as well as with deaths from accidents and violence with alcohol as a contributing cause. The observed association between living alone and alcohol-related mortality was robust to adjustment for multiple indicators of socioeconomic position.

### Strengths and Weaknesses

Our study was based on a large population-based sample of Finns, and we took into account oversampling of deaths in all analyses; the findings are therefore likely to be generalisable to the Finnish population aged 15–79 y. With information on both underlying and contributory causes of death, based on autopsy in most cases, our mortality data were likely to capture a full range of alcohol-related deaths. We used living arrangements rather than marital status as an index of social relationships because living arrangements may reflect social relationships more accurately, especially given the increasing proportion of persons recorded as never married, divorced, or widowed but still living with a partner [18]. According to our sensitivity analysis, bias due to coding artefacts is an unlikely explanation for our results. This is in agreement with the highly ranked reliability and accuracy of the Finnish death register in international comparisons [16].

There are a few caveats to the results reported here. First, as longitudinal data on the history of alcohol consumption and living arrangements were not available in this dataset, we cannot know the extent to which living alone might be a cause or a consequence of alcohol abuse. However, a greater increase in fatal liver disease among individuals living alone after the alcohol price reduction strongly suggests that persons living alone are at least more vulnerable to the adverse effects of higher alcohol availability. Second, the before–after design used here is not optimal in taking into account effects of general secular trends in alcohol-related mortality or differences in latency periods between alcohol-related diseases. This limitation could have led to under- or overestimation of the effects of the price reduction.

### Comparison with Previous Studies

Our findings are consistent with several previous studies on marital status and mortality. In the 18-y follow-up of 18,403 men aged 40–64 y participating in the Whitehall study, single men compared to married men had a risk ratio of 1.9 for violent and accidental deaths (many of which can be assumed to be alcohol related) [19]. A Swedish study on premature mortality found that lone non-custodial fathers and lone childless men had a greater a risk of death from addiction (alcohol and narcotics related, combined) than cohabiting custodial fathers [20]. A Finnish study showed a 3-to 5-fold excess in overall alcohol-related mortality among unmarried men and women aged 30–64 y compared with married men and women [6].

We found gender differences in the association between living alone and alcohol-related mortality, with greater risk ratios in all alcohol-related cause-of-death categories among men than among women. This is in agreement with an analysis of 16 developed countries in which mortality of unmarried men (relative to married men) exceeded that of unmarried women [5]. Earlier studies have also indicated that men are more dependent on spouse where health disparities across marital status are concerned [21]–[23].

### Meaning of the Study

The largest relative excess mortality among those living alone was from deaths from alcohol dependence and poisoning, other alcohol-related diseases, and alcohol-attributable cardiovascular diseases. Alcohol dependence or alcoholism as a cause of death represents typically the endpoint of a long-term severe degree of alcohol abuse [24], and acute alcohol poisoning is often a complication of chronic alcoholism (a casual drinker usually does not reach a lethal concentration of ethyl alcohol in blood) [25]. The amount and duration of alcohol use that results in alcoholic cardiomyopathy is not precisely established [26], but some studies suggest that alcoholic patients with heart failure have a mean daily consumption of over 240 g of alcohol over an average of 16 y [27].

The excess increase in alcohol-related mortality after the price reduction among persons living alone was mainly attributable to an increase in liver disease. The latency period for liver cirrhosis, the major category of liver disease mortality, is long, up to 20 y of excessive drinking [28]. This implies that a great proportion of these excess deaths after the price reduction were among individuals who had been alcohol abusers long before the price reduction. Absence of various supportive or protective mechanisms related to marriage and cohabiting among those living alone may have contributed to their increased alcohol consumption and death after the price reduction.

Living alone was also associated with mortality from accidents and violence, with alcohol as a contributing cause, but we did not observe meaningful changes in this mortality or the relative risk after the price reduction. An accidental fall or a transport accident may happen even to a person who is not a chronic alcohol abuser, and a study in the US found that the group at highest risk of death from external causes consisted of drinkers who drank infrequently, once a month or less, and usually five or more drinks at a time [29]. Alcohol tolerance was hypothesized to have an influence on the risk of injury. Another study, however, found that the risk was highest among those who had the highest number of heavy drinking occasions [30]. The observation that the change in mortality from these causes was marginal after the price change suggests that increased excessive drinking after the price reduction was mainly confined to those who had a long history of abusing alcohol already before the price reduction. Moreover, it appears that increased availability of alcohol has not necessarily increased sporadic binge drinking occasions among those who are not heavy drinkers, regardless of whether they are living alone or married or cohabiting.

### Implications and Future Research

Although European guidelines on cardiovascular disease prevention in clinical practice count social isolation as a risk factor for coronary heart disease [31], the idea that a lack of social relationships is a risk factor for death is still not widely recognised by health professionals, policy makers, or the public [1]. This natural experimental study suggests that a lack of social relationships, for which living alone is a relevant indicator, should be regarded as a potential risk marker for death from alcohol-related causes.

Further longitudinal research is needed to confirm the generalisability of our findings to other countries with different alcohol cultures (e.g., Mediterranean wine culture) and to identify selective and causal processes underlying the association between living alone and alcohol abuse.

## Supporting Information

Table S1 **Relative mortality from selected causes of death for living alone versus married or cohabiting in men and women aged 15–79 y before (2000–2003) and after (2004–2007) the alcohol price reduction.**
(DOC)

Table S2 **Relative mortality from selected causes of death (alcohol-related included) for living alone versus married or cohabiting in men aged 15–79 y before (2000–2003) and after (2004–2007) the alcohol price reduction.**
(DOC)

Table S3 **Relative mortality from selected causes of death (alcohol-related included) for living alone versus married or cohabiting in women aged 15–79 y before (2000–2003) and after (2004–2007) the alcohol price reduction.**
(DOC)

Table S4 **Relative mortality from selected causes of death (alcohol-related excluded) for living alone versus married or cohabiting in men aged 15–79 y before (2000–2003) and after (2004–2007) the alcohol price reduction.**
(DOC)

Table S5 **Relative mortality from selected causes of death (alcohol-related excluded) for living alone versus married or cohabiting in women aged 15–79 y before (2000–2003) and after (2004–2007) the alcohol price reduction.**
(DOC)

Table S6 **Relative alcohol-related mortality for living alone versus married or cohabiting in men and women aged 15–79 y before (2000–2003) and after (2004–2007) the alcohol price reduction.** Overall model in which sex is included as an additional independent variable.
(DOC)

Table S7 **Relative alcohol-related mortality for living alone versus married or cohabiting in men and women aged 15–79 y before (2000–2003) and after (2004–2007) the alcohol price reduction.** Adjusted for continuous age term.
(DOC)

Table S8 **Relative alcohol-related mortality for living alone versus married or cohabiting in men and women aged 15–79 y before (2000–2003) and after (2004–2007) the alcohol price reduction.** Adjusted for squared age term.
(DOC)

Table S9 **Relative alcohol-related mortality for living alone versus married or cohabiting in men and women aged 15–79 y before (2000–2003) and after (2004–2007) the alcohol price reduction.** Adjusted for continuous age and squared age term.
(DOC)

## Abbreviations

CI: confidence interval
ICD-10: *International Statistical Classification of Diseases and Related Health Problems, 10th revision*
