# Supplementary material for: Living Alone and Alcohol-Related Mortality: A Population-Based Cohort Study from Finland
Source: PLoS Med. 2011 Sep 20;8(9):e1001094. doi: 10.1371/journal.pmed.1001094 (PMC3176753; doi:10.1371/journal.pmed.1001094)
Supplement: Table S1 — Relative mortality from selected causes of death for living alone versus married or cohabiting in men and women aged 15–79 y before (2000–2003) and after (2004–2007) the alcohol price reduction. (DOC) [file pmed.1001094.s001.doc]

|  | |  |  | Risk ratios for living alone vs. married or cohabiting | | | | | | |  | | | | | | | | | |
| --- | --- | --- | --- | --- | --- | --- | --- | --- | --- | --- | --- | --- | --- | --- | --- | --- | --- | --- | --- | --- |
|  | |  |  | Model 1 | | Model 2 | | Model 3 | | |  | | | | | | | | | |
|  | | Deaths^b^ | Rate^c^ | RR | 95% CI | RR | 95% CI | RR | 95% CI | |  | | | | | | | | | |
| MEN, BEFORE | |  |  |  |  |  |  |  |  | |  | | | | | | | | | |
| Married or cohabiting | | 7475 | 196.2 | 1.00 |  | 1.00 |  | 1.00 |  | |  | | | | | | | | | |
| Living alone | | 6779 | 664.9 | 3.37 | 3.25-3.49 | 3.22 | 3.11-3.33 | 2.72 | 2.62-2.82 | |  | | | | | | | | | |
| MEN, AFTER | |  |  |  |  |  |  |  |  | |  | | | | | | | | | |
| Married or cohabiting | | 5853 | 193.8 | 1.00 |  | 1.00 |  | 1.00 |  | |  | | | | | | | | | |
| Living alone | | 8287 | 719.6 | 3.65 | 3.52-3.79 | 3.48 | 3.35-3.61 | 2.83 | 2.73-2.94 | |  | | | | | | | | | |
| P value^d^ | |  |  | 0.001 |  | 0.002 |  | 0.005 |  | |  | | | | | | | | | |
| WOMEN, BEFORE | |  |  |  |  |  |  |  |  | |  | | | | | | | | | |
| Married or cohabiting | | 3096 | 103.5 | 1.00 |  | 1.00 |  | 1.00 |  | |  | | | | | | | | | |
| Living alone | | 3040 | 184.6 | 1.69 | 1.59-1.79 | 1.68 | 1.59-1.78 | 1.81 | 1.71-1.92 | |  | | | | | | | | | |
| WOMEN, AFTER | |  |  |  |  |  |  |  |  | |  | | | | | | | | | |
| Married or cohabiting | | 2505 | 102.0 | 1.00 |  | 1.00 |  | 1.00 |  | |  | | | | | | | | | |
| Living alone | | 3212 | 197.2 | 1.79 | 1.68-1.90 | 1.76 | 1.65-1.87 | 1.86 | 1.75-1.98 | |  | | | | | | | | | |
| P value^d^ | |  |  | 0.988 |  | 0.784 |  | 0.466 |  | |  | | | | | | | | | |
|  | ^a^ Cause of death categories include gastro-intestinal causes, neuro-psychiatric causes, intentional injuries, | | | | | | | | | | | | | | | | | | | |
|  | non-intentional injuries, non-specific causes, and all alcohol-related causes. | | | | | | | | | | | | | | | | |  |  |  |
|  | ^b^ Numbers of deaths are those observed in the original sample. | | | | | | | | |  | |  |  |  |  |  |  |  |  |  |
|  | ^c^ Mortality rates (deaths per 100,000) adjusted for age. | | | | | | | | |  | |  |  |  |  |  |  |  |  |  |
|  | Model 1: adjusted for age. | | | | | | | | |  | |  |  |  |  |  |  |  |  |  |
|  | Model 2: adjusted for age, education and social class. | | | | | | | | |  | |  |  |  |  |  |  |  |  |  |
|  | Model 3: adjusted for age, education, social class and income. | | | | | | | | |  | |  |  |  |  |  |  |  |  |  |
|  | ^d^ P value for change in difference in excess mortality for those living alone compared to married and cohabiting persons. | | | | | | | | | | | | | | | | | | | |

| **Table S1.** Relative mortality from selected causes of death^a^ for living alone vs. married and cohabiting in men and women aged 15-79 years before (2000-2003) and after (2004-2007) the price reduction. |
| --- |
