# Supplementary material for: Living Alone and Alcohol-Related Mortality: A Population-Based Cohort Study from Finland
Source: PLoS Med. 2011 Sep 20;8(9):e1001094. doi: 10.1371/journal.pmed.1001094 (PMC3176753; doi:10.1371/journal.pmed.1001094)
Supplement: Table S2 — Relative mortality from selected causes of death (alcohol-related included) for living alone versus married or cohabiting in men aged 15–79 y before (2000–2003) and after (2004–2007) the alcohol price reduction. (DOC) [file pmed.1001094.s002.doc]

|  | |  |  | Risk ratios for living alone vs. married or cohabiting | | | | | |  | | | | | | | | | | |
| --- | --- | --- | --- | --- | --- | --- | --- | --- | --- | --- | --- | --- | --- | --- | --- | --- | --- | --- | --- | --- |
|  | |  |  | Model 1 | | Model 2 | | Model 3 | |  | | | | | | | | | | |
| Cause of death | | Deaths^a^ | Rate^b^ | RR | 95% CI | RR | 95% CI | RR | 95% CI |  | | | | | | | | | | |
| BEFORE | |  |  |  |  |  |  |  |  |  | | | | | | | | | | |
| Gastro-intestinal causes | |  |  |  |  |  |  |  |  |  | | | | | | | | | | |
| Married or cohabiting | | 2032 | 53.7 | 1.00 |  | 1.00 |  | 1.00 |  |  | | | | | | | | | | |
| Living alone | | 900 | 89.5 | 1.64 | 1.51-1.78 | 1.60 | 1.48-1.73 | 1.50 | 1.39-1.63 |  | | | | | | | | | | |
| Neuro-psychiatric causes | |  |  |  |  |  |  |  |  |  | | | | | | | | | | |
| Married or cohabiting | | 1054 | 28.3 | 1.00 |  | 1.00 |  | 1.00 |  |  | | | | | | | | | | |
| Living alone | | 290 | 28.0 | 0.99 | 0.87-1.13 | 0.98 | 0.86-1.12 | 0.92 | 0.81-1.05 |  | | | | | | | | | | |
| Intentional injuries | |  |  |  |  |  |  |  |  |  | | | | | | | | | | |
| Married or cohabiting | | 1108 | 26.1 | 1.00 |  | 1.00 |  | 1.00 |  |  | | | | | | | | | | |
| Living alone | | 1254 | 114.3 | 3.83 | 3.53-4.16 | 3.58 | 3.29-3.89 | 3.03 | 2.78-3.30 |  | | | | | | | | | | |
| Non-intentional injuries | |  |  |  |  |  |  |  |  |  | | | | | | | | | | |
| Married or cohabiting | | 1514 | 40.0 | 1.00 |  | 1.00 |  | 1.00 |  |  | | | | | | | | | | |
| Living alone | | 1482 | 142.9 | 3.60 | 3.35-3.87 | 3.41 | 3.17-3.67 | 2.95 | 2.74-3.18 |  | | | | | | | | | | |
| Non-specific causes | |  |  |  |  |  |  |  |  |  | | | | | | | | | | |
| Married or cohabiting | | 20 | 0.5 | 1.00 |  | 1.00 |  | 1.00 |  |  | | | | | | | | | | |
| Living alone | | 154 | 14.9 | 27.45 | 17.2-43.8 | 26.22 | 16.4-41.9 | 19.86 | 12.4-31.8 |  | | | | | | | | | | |
| AFTER | |  |  |  |  |  |  |  |  |  | | | | | | | | | | |
| Gastro-intestinal causes | |  |  |  |  |  |  |  |  |  | | | | | | | | | | |
| Married or cohabiting | | 1534 | 43.8 | 1.00 |  | 1.00 |  | 1.00 |  |  | | | | | | | | | | |
| Living alone | | 1062 | 92.6 | 1.82 | 1.67-1.99 | 1.78 | 1.63-1.94 | 1.64 | 1.51-1.79 |  | | | | | | | | | | |
| P value^c^ | |  |  |  | 0.073 |  | 0.082 |  | 0.128 |  | | | | | | | | | | |
| Neuro-psychiatric causes | |  |  |  |  |  |  |  |  |  | | | | | | | | | | |
| Married or cohabiting | | 879 | 28.7 | 1.00 |  | 1.00 |  | 1.00 |  |  | | | | | | | | | | |
| Living alone | | 357 | 30.4 | 1.06 | 0.92-1.22 | 1.06 | 0.92-1.22 | 0.98 | 0.85-1.13 |  | | | | | | | | | | |
| P value^c^ | |  |  |  | 0.498 |  | 0.502 |  | 0.613 |  | | | | | | | | | | |
| Intentional injuries | |  |  |  |  |  |  |  |  |  | | | | | | | | | | |
| Married or cohabiting | | 724 | 25.5 | 1.00 |  | 1.00 |  | 1.00 |  |  | | | | | | | | | | |
| Living alone | | 1168 | 96.3 | 3.76 | 3.40-4.15 | 3.50 | 3.16-3.87 | 2.83 | 2.56-3.14 |  | | | | | | | | | | |
| P value^c^ | |  |  |  | 0.656 |  | 0.561 |  | 0.315 |  | | | | | | | | | | |
| Non-intentional injuries | |  |  |  |  |  |  |  |  |  | | | | | | | | | | |
| Married or cohabiting | | 1168 | 39.5 | 1.00 |  | 1.00 |  | 1.00 |  |  | | | | | | | | | | |
| Living alone | | 1730 | 146.2 | 3.65 | 3.37-3.95 | 3.42 | 3.16-3.71 | 2.80 | 2.59-3.04 |  | | | | | | | | | | |
| P value^c^ | |  |  |  | 0.717 |  | 0.796 |  | 0.853 |  | | | | | | | | | | |
| Non-specific causes | |  |  |  |  |  |  |  |  |  | | | | | | | | | | |
| Married or cohabiting | | 15 | 0.5 | 1.00 |  | 1.00 |  | 1.00 |  |  | | | | | | | | | | |
| Living alone | | 201 | 17.8 | 34.15 | 20.158.1 | 32.93 | 19.3-56.2 | 25.11 | 14.7-42.8 |  | | | | | | | | | | |
| P value^c^ | |  |  |  | 0.578 |  | 0.587 |  | 0.667 |  | | | | | | | | | | |
|  | ^a^ Numbers of deaths are those observed in the original sample. | | | | | | | | | |  |  |  |  |  |  |  |  |  |  |
|  | ^b^ Mortality rates (deaths per 100,000) adjusted for age. | | | | | | | | | |  |  |  |  |  |  |  |  |  |  |
|  | Model 1: adjusted for age. | | | | | | | | | |  |  |  |  |  |  |  |  |  |  |
|  | Model 2: adjusted for age, education and social class. | | | | | | | | | |  |  |  |  |  |  |  |  |  |  |
|  | Model 3: adjusted for age, education, social class and income. | | | | | | | | | |  |  |  |  |  |  |  |  |  |  |
|  | ^c^ P value for change in difference in excess mortality for those living alone compared to married and cohabiting persons. | | | | | | | | | |  |  |  |  |  |  |  |  |  |  |

| **Table S2.** Relative mortality from selected causes of death (alcohol-related included) for living alone vs. married and cohabiting in men aged 15-79 years before (2000-2003) and after (2004-2007) the price reduction. |
| --- |
