# Supplementary material for: Living Alone and Alcohol-Related Mortality: A Population-Based Cohort Study from Finland
Source: PLoS Med. 2011 Sep 20;8(9):e1001094. doi: 10.1371/journal.pmed.1001094 (PMC3176753; doi:10.1371/journal.pmed.1001094)
Supplement: Table S3 — Relative mortality from selected causes of death (alcohol-related included) for living alone versus married or cohabiting in women aged 15–79 y before (2000–2003) and after (2004–2007) the alcohol price reduction. (DOC) [file pmed.1001094.s003.doc]

|  | |  |  | Risk ratios for living alone vs. married or cohabiting | | | | | |  | | | | | | | | | | |
| --- | --- | --- | --- | --- | --- | --- | --- | --- | --- | --- | --- | --- | --- | --- | --- | --- | --- | --- | --- | --- |
|  | |  |  | Model 1 | | Model 2 | | Model 3 | |  | | | | | | | | | | |
| Cause of death | | Deaths^a^ | Rate^b^ | RR | 95% CI | RR | 95% CI | RR | 95% CI |  | | | | | | | | | | |
| BEFORE | |  |  |  |  |  |  |  |  |  | | | | | | | | | | |
| Gastro-intestinal causes | |  |  |  |  |  |  |  |  |  | | | | | | | | | | |
| Married or cohabiting | | 1055 | 38.0 | 1.00 |  | 1.00 |  | 1.00 |  |  | | | | | | | | | | |
| Living alone | | 1148 | 53.7 | 1.37 | 1.25-1.50 | 1.37 | 1.25-1.50 | 1.45 | 1.32-1.59 |  | | | | | | | | | | |
| Neuro-psychiatric causes | |  |  |  |  |  |  |  |  |  | | | | | | | | | | |
| Married or cohabiting | | 622 | 25.4 | 1.00 |  | 1.00 |  | 1.00 |  |  | | | | | | | | | | |
| Living alone | | 427 | 18.8 | 0.72 | 0.63-0.82 | 0.72 | 0.63-0.82 | 0.81 | 0.71-0.93 |  | | | | | | | | | | |
| Intentional injuries | |  |  |  |  |  |  |  |  |  | | | | | | | | | | |
| Married or cohabiting | | 389 | 12.0 | 1.00 |  | 1.00 |  | 1.00 |  |  | | | | | | | | | | |
| Living alone | | 405 | 36.8 | 3.01 | 2.61-3.48 | 2.93 | 2.54-3.38 | 2.94 | 2.55-3.40 |  | | | | | | | | | | |
| Non-intentional injuries | |  |  |  |  |  |  |  |  |  | | | | | | | | | | |
| Married or cohabiting | | 494 | 15.9 | 1.00 |  | 1.00 |  | 1.00 |  |  | | | | | | | | | | |
| Living alone | | 533 | 31.8 | 2.05 | 1.79-2.35 | 2.03 | 1.77-2.32 | 2.14 | 1.88-2.45 |  | | | | | | | | | | |
| Non-specific causes | |  |  |  |  |  |  |  |  |  | | | | | | | | | | |
| Married or cohabiting | | 32 | 0.9 | 1.00 |  | 1.00 |  | 1.00 |  |  | | | | | | | | | | |
| Living alone | | 81 | 6.6 | 6.42 | 4.22-9.77 | 6.44 | 4.22-9.81 | 6.77 | 4.44-10.3 |  | | | | | | | | | | |
| AFTER | |  |  |  |  |  |  |  |  |  | | | | | | | | | | |
| Gastro-intestinal causes | |  |  |  |  |  |  |  |  |  | | | | | | | | | | |
| Married or cohabiting | | 825 | 35.5 | 1.00 |  | 1.00 |  | 1.00 |  |  | | | | | | | | | | |
| Living alone | | 1057 | 50.0 | 1.35 | 1.21-1.49 | 1.34 | 1.21-1.49 | 1.42 | 1.29-1.58 |  | | | | | | | | | | |
| P value^c^ | |  |  |  | 0.279 |  | 0.237 |  | 0.192 |  | | | | | | | | | | |
| Neuro-psychiatric causes | |  |  |  |  |  |  |  |  |  | | | | | | | | | | |
| Married or cohabiting | | 492 | 24.0 | 1.00 |  | 1.00 |  | 1.00 |  |  | | | | | | | | | | |
| Living alone | | 431 | 19.9 | 0.80 | 0.69-0.94 | 0.80 | 0.69-0.93 | 0.90 | 0.77-1.05 |  | | | | | | | | | | |
| P value^c^ | |  |  |  | 0.514 |  | 0.530 |  | 0.608 |  | | | | | | | | | | |
| Intentional injuries | |  |  |  |  |  |  |  |  |  | | | | | | | | | | |
| Married or cohabiting | | 284 | 9.7 | 1.00 |  | 1.00 |  | 1.00 |  |  | | | | | | | | | | |
| Living alone | | 351 | 29.9 | 2.43 | 2.04-2.89 | 2.30 | 1.94-2.74 | 2.27 | 1.91-2.71 |  | | | | | | | | | | |
| P value^c^ | |  |  |  | 0.054 |  | 0.046 |  | 0.030 |  | | | | | | | | | | |
| Non-intentional injuries | |  |  |  |  |  |  |  |  |  | | | | | | | | | | |
| Married or cohabiting | | 412 | 15.9 | 1.00 |  | 1.00 |  | 1.00 |  |  | | | | | | | | | | |
| Living alone | | 582 | 36.8 | 2.28 | 1.98-2.63 | 2.21 | 1.92-2.55 | 2.27 | 1.98-2.61 |  | | | | | | | | | | |
| P value^c^ | |  |  |  | 0.950 |  | 0.974 |  | 0.840 |  | | | | | | | | | | |
| Non-specific causes | |  |  |  |  |  |  |  |  |  | | | | | | | | | | |
| Married or cohabiting | | 22 | 0.8 | 1.00 |  | 1.00 |  | 1.00 |  |  | | | | | | | | | | |
| Living alone | | 98 | 7.9 | 7.68 | 4.72-12.5 | 7.80 | 4.80-12.7 | 8.04 | 4.94-13.1 |  | | | | | | | | | | |
| P value^c^ | |  |  |  | 0.651 |  | 0.647 |  | 0.724 |  | | | | | | | | | | |
|  | ^a^ Numbers of deaths are those observed in the original sample. | | | | | | | | | |  |  |  |  |  |  |  |  |  |  |
|  | ^b^ Mortality rates (deaths per 100,000) adjusted for age. | | | | | | | | | |  |  |  |  |  |  |  |  |  |  |
|  | Model 1: adjusted for age. | | | | | | | | | |  |  |  |  |  |  |  |  |  |  |
|  | Model 2: adjusted for age, education and social class. | | | | | | | | | |  |  |  |  |  |  |  |  |  |  |
|  | Model 3: adjusted for age, education, social class and income. | | | | | | | | | |  |  |  |  |  |  |  |  |  |  |
|  | ^c^ P value for change in difference in excess mortality for those living alone compared to married and cohabiting persons. | | | | | | | | | |  |  |  |  |  |  |  |  |  |  |

| **Table S3.** Relative mortality from selected causes of death (alcohol-related included) for living alone vs. married and cohabiting in women aged 15-79 years before (2000-2003) and after (2004-2007) the price reduction. |
| --- |
