# Supplementary material for: Living Alone and Alcohol-Related Mortality: A Population-Based Cohort Study from Finland
Source: PLoS Med. 2011 Sep 20;8(9):e1001094. doi: 10.1371/journal.pmed.1001094 (PMC3176753; doi:10.1371/journal.pmed.1001094)
Supplement: Table S4 — Relative mortality from selected causes of death (alcohol-related excluded) for living alone versus married or cohabiting in men aged 15–79 y before (2000–2003) and after (2004–2007) the alcohol price reduction. (DOC) [file pmed.1001094.s004.doc]

|  | |  |  | Risk ratios for living alone vs. married or cohabiting | | | | | |  | | | | | | | | | | |
| --- | --- | --- | --- | --- | --- | --- | --- | --- | --- | --- | --- | --- | --- | --- | --- | --- | --- | --- | --- | --- |
|  | |  |  | Model 1 | | Model 2 | | Model 3 | |  | | | | | | | | | | |
| Cause of death | | Deaths^a^ | Rate^b^ | RR | 95% CI | RR | 95% CI | RR | 95% CI |  | | | | | | | | | | |
| BEFORE | |  |  |  |  |  |  |  |  |  | | | | | | | | | | |
| Gastro-intestinal causes | |  |  |  |  |  |  |  |  |  | | | | | | | | | | |
| Married or cohabiting | | 1985 | 52.4 | 1.00 |  | 1.00 |  | 1.00 |  |  | | | | | | | | | | |
| Living alone | | 816 | 80.8 | 1.51 | 1.40-1.65 | 1.48 | 1.36-1.61 | 1.41 | 1.29-1.53 |  | | | | | | | | | | |
| Neuro-psychiatric causes | |  |  |  |  |  |  |  |  |  | | | | | | | | | | |
| Married or cohabiting | | 1047 | 28.2 | 1.00 |  | 1.00 |  | 1.00 |  |  | | | | | | | | | | |
| Living alone | | 266 | 25.6 | 0.91 | 0.79-1.05 | 0.90 | 0.79-1.04 | 0.85 | 0.74-0.98 |  | | | | | | | | | | |
| Intentional injuries | |  |  |  |  |  |  |  |  |  | | | | | | | | | | |
| Married or cohabiting | | 636 | 16.7 | 1.00 |  | 1.00 |  | 1.00 |  |  | | | | | | | | | | |
| Living alone | | 675 | 62.2 | 3.69 | 3.31-4.11 | 3.47 | 3.11-3.88 | 3.00 | 2.68-3.36 |  | | | | | | | | | | |
| Non-intentional injuries | |  |  |  |  |  |  |  |  |  | | | | | | | | | | |
| Married or cohabiting | | 983 | 26.1 | 1.00 |  | 1.00 |  | 1.00 |  |  | | | | | | | | | | |
| Living alone | | 669 | 63.6 | 2.54 | 2.30-2.80 | 2.41 | 2.18-2.66 | 2.20 | 1.99-2.44 |  | | | | | | | | | | |
| Non-specific causes | |  |  |  |  |  |  |  |  |  | | | | | | | | | | |
| Married or cohabiting | | 20 | 0.5 | 1.00 |  | 1.00 |  | 1.00 |  |  | | | | | | | | | | |
| Living alone | | 154 | 14.9 | 27.45 | 17.2-43.8 | 26.22 | 16.4-41.9 | 19.86 | 12.4-31.8 |  | | | | | | | | | | |
| AFTER | |  |  |  |  |  |  |  |  |  | | | | | | | | | | |
| Gastro-intestinal causes | |  |  |  |  |  |  |  |  |  | | | | | | | | | | |
| Married or cohabiting | | 1491 | 48.4 | 1.00 |  | 1.00 |  | 1.00 |  |  | | | | | | | | | | |
| Living alone | | 924 | 80.3 | 1.65 | 1.51-1.81 | 1.61 | 1.47-1.77 | 1.51 | 1.38-1.66 |  | | | | | | | | | | |
| P value^c^ | |  |  |  | 0.178 |  | 0.195 |  | 0.261 |  | | | | | | | | | | |
| Neuro-psychiatric causes | |  |  |  |  |  |  |  |  |  | | | | | | | | | | |
| Married or cohabiting | | 871 | 28.5 | 1.00 |  | 1.00 |  | 1.00 |  |  | | | | | | | | | | |
| Living alone | | 328 | 28.0 | 0.98 | 0.85-1.13 | 0.98 | 0.85-1.14 | 0.92 | 0.80-1.06 |  | | | | | | | | | | |
| P value^c^ | |  |  |  | 0.453 |  | 0.456 |  | 0.550 |  | | | | | | | | | | |
| Intentional injuries | |  |  |  |  |  |  |  |  |  | | | | | | | | | | |
| Married or cohabiting | | 372 | 12.9 | 1.00 |  | 1.00 |  | 1.00 |  |  | | | | | | | | | | |
| Living alone | | 590 | 49.0 | 3.85 | 3.36-4.42 | 3.66 | 3.18-4.21 | 3.00 | 2.60-3.46 |  | | | | | | | | | | |
| P value^c^ | |  |  |  | 0.756 |  | 0.807 |  | 0.972 |  | | | | | | | | | | |
| Non-intentional injuries | |  |  |  |  |  |  |  |  |  | | | | | | | | | | |
| Married or cohabiting | | 712 | 24.1 | 1.00 |  | 1.00 |  | 1.00 |  |  | | | | | | | | | | |
| Living alone | | 678 | 55.9 | 2.40 | 2.14-2.69 | 2.28 | 2.04-2.56 | 1.99 | 1.78-2.24 |  | | | | | | | | | | |
| P value^c^ | |  |  |  | 0.529 |  | 0.488 |  | 0.371 |  | | | | | | | | | | |
| Non-specific causes | |  |  |  |  |  |  |  |  |  | | | | | | | | | | |
| Married or cohabiting | | 14 | 0.5 | 1.00 |  | 1.00 |  | 1.00 |  |  | | | | | | | | | | |
| Living alone | | 196 | 17.4 | 35.42 | 20.5-61.3 | 34.21 | 19.7-59.4 | 26.02 | 15.0-45.1 |  | | | | | | | | | | |
| P value^c^ | |  |  |  | 0.520 |  | 0.528 |  | 0.604 |  | | | | | | | | | | |
|  | ^a^ Numbers of deaths are those observed in the original sample. | | | | | | | | | |  |  |  |  |  |  |  |  |  |  |
|  | ^b^ Mortality rates (deaths per 100,000) adjusted for age. | | | | | | | | | |  |  |  |  |  |  |  |  |  |  |
|  | Model 1: adjusted for age. | | | | | | | | | |  |  |  |  |  |  |  |  |  |  |
|  | Model 2: adjusted for age, education and social class. | | | | | | | | | |  |  |  |  |  |  |  |  |  |  |
|  | Model 3: adjusted for age, education, social class and income. | | | | | | | | | |  |  |  |  |  |  |  |  |  |  |
|  | ^c^ P value for change in difference in excess mortality for those living alone compared to married and cohabiting persons. | | | | | | | | | |  |  |  |  |  |  |  |  |  |  |

| **Table S4.** Relative mortality from selected causes of death (alcohol-related excluded) for living alone vs. married and cohabiting in men aged 15-79 years before (2000-2003) and after (2004-2007) the price reduction. |
| --- |
