# Supplementary material for: Living Alone and Alcohol-Related Mortality: A Population-Based Cohort Study from Finland
Source: PLoS Med. 2011 Sep 20;8(9):e1001094. doi: 10.1371/journal.pmed.1001094 (PMC3176753; doi:10.1371/journal.pmed.1001094)
Supplement: Table S5 — Relative mortality from selected causes of death (alcohol-related excluded) for living alone versus married or cohabiting in women aged 15–79 y before (2000–2003) and after (2004–2007) the alcohol price reduction. (DOC) [file pmed.1001094.s005.doc]

|  | |  |  | Risk ratios for living alone vs. married or cohabiting | | | | | |  | | | | | | | | | | |
| --- | --- | --- | --- | --- | --- | --- | --- | --- | --- | --- | --- | --- | --- | --- | --- | --- | --- | --- | --- | --- |
|  | |  |  | Model 1 | | Model 2 | | Model 3 | |  | | | | | | | | | | |
| Cause of death | | Deaths^a^ | Rate^b^ | RR | 95% CI | RR | 95% CI | RR | 95% CI |  | | | | | | | | | | |
| BEFORE | |  |  |  |  |  |  |  |  |  | | | | | | | | | | |
| Gastro-intestinal causes | |  |  |  |  |  |  |  |  |  | | | | | | | | | | |
| Married or cohabiting | | 1039 | 37.5 | 1.00 |  | 1.00 |  | 1.00 |  |  | | | | | | | | | | |
| Living alone | | 1129 | 52.4 | 1.35 | 1.24-1.48 | 1.36 | 1.24-1.49 | 1.43 | 1.31-1.57 |  | | | | | | | | | | |
| Neuro-psychiatric causes | |  |  |  |  |  |  |  |  |  | | | | | | | | | | |
| Married or cohabiting | | 619 | 25.3 | 1.00 |  | 1.00 |  | 1.00 |  |  | | | | | | | | | | |
| Living alone | | 423 | 18.2 | 0.71 | 0.62-0.81 | 0.71 | 0.62-0.81 | 0.79 | 0.69-0.91 |  | | | | | | | | | | |
| Intentional injuries | |  |  |  |  |  |  |  |  |  | | | | | | | | | | |
| Married or cohabiting | | 266 | 6.9 | 1.00 |  | 1.00 |  | 1.00 |  |  | | | | | | | | | | |
| Living alone | | 293 | 25.4 | 3.05 | 2.57-3.63 | 2.99 | 2.52-3.56 | 3.02 | 2.55-3.59 |  | | | | | | | | | | |
| Non-intentional injuries | |  |  |  |  |  |  |  |  |  | | | | | | | | | | |
| Married or cohabiting | | 349 | 12.0 | 1.00 |  | 1.00 |  | 1.00 |  |  | | | | | | | | | | |
| Living alone | | 423 | 22.6 | 2.05 | 1.75-2.41 | 2.04 | 1.74-2.39 | 2.17 | 1.85-2.53 |  | | | | | | | | | | |
| Non-specific causes | |  |  |  |  |  |  |  |  |  | | | | | | | | | | |
| Married or cohabiting | | 32 | 0.9 | 1.00 |  | 1.00 |  | 1.00 |  |  | | | | | | | | | | |
| Living alone | | 81 | 6.6 | 6.42 | 4.22-9.77 | 6.44 | 4.22-9.81 | 6.77 | 4.44-10.3 |  | | | | | | | | | | |
| AFTER | |  |  |  |  |  |  |  |  |  | | | | | | | | | | |
| Gastro-intestinal causes | |  |  |  |  |  |  |  |  |  | | | | | | | | | | |
| Married or cohabiting | | 802 | 34.6 | 1.00 |  | 1.00 |  | 1.00 |  |  | | | | | | | | | | |
| Living alone | | 1029 | 48.0 | 1.34 | 1.20-1.48 | 1.33 | 1.20-1.48 | 1.41 | 1.27-1.56 |  | | | | | | | | | | |
| P value^c^ | |  |  |  | 0.350 |  | 0.303 |  | 0.253 |  | | | | | | | | | | |
| Neuro-psychiatric causes | |  |  |  |  |  |  |  |  |  | | | | | | | | | | |
| Married or cohabiting | | 489 | 24.0 | 1.00 |  | 1.00 |  | 1.00 |  |  | | | | | | | | | | |
| Living alone | | 423 | 19.4 | 0.78 | 0.67-0.91 | 0.78 | 0.67-0.91 | 0.87 | 0.74-1.01 |  | | | | | | | | | | |
| P value^c^ | |  |  |  | 0.607 |  | 0.624 |  | 0.702 |  | | | | | | | | | | |
| Intentional injuries | |  |  |  |  |  |  |  |  |  | | | | | | | | | | |
| Married or cohabiting | | 167 | 5.9 | 1.00 |  | 1.00 |  | 1.00 |  |  | | | | | | | | | | |
| Living alone | | 234 | 18.9 | 2.63 | 2.11-3.28 | 2.53 | 2.03-3.16 | 2.52 | 2.02-3.14 |  | | | | | | | | | | |
| P value^c^ | |  |  |  | 0.287 |  | 0.276 |  | 0.220 |  | | | | | | | | | | |
| Non-intentional injuries | |  |  |  |  |  |  |  |  |  | | | | | | | | | | |
| Married or cohabiting | | 282 | 11.4 | 1.00 |  | 1.00 |  | 1.00 |  |  | | | | | | | | | | |
| Living alone | | 378 | 20.1 | 1.91 | 1.60-2.27 | 1.88 | 1.58-2.23 | 1.93 | 1.63-2.29 |  | | | | | | | | | | |
| P value^c^ | |  |  |  | 0.169 |  | 0.152 |  | 0.124 |  | | | | | | | | | | |
| Non-specific causes | |  |  |  |  |  |  |  |  |  | | | | | | | | | | |
| Married or cohabiting | | 22 | 0.7 | 1.00 |  | 1.00 |  | 1.00 |  |  | | | | | | | | | | |
| Living alone | | 94 | 7.6 | 7.42 | 4.55-12.1 | 7.53 | 4.62-12.3 | 7.77 | 4.77-12.7 |  | | | | | | | | | | |
| P value^c^ | |  |  |  | 0.721 |  | 0.717 |  | 0.796 |  | | | | | | | | | | |
|  | ^a^ Numbers of deaths are those observed in the original sample. | | | | | | | | | |  |  |  |  |  |  |  |  |  |  |
|  | ^b^ Mortality rates (deaths per 100,000) adjusted for age. | | | | | | | | | |  |  |  |  |  |  |  |  |  |  |
|  | Model 1: adjusted for age. | | | | | | | | | |  |  |  |  |  |  |  |  |  |  |
|  | Model 2: adjusted for age, education and social class. | | | | | | | | | |  |  |  |  |  |  |  |  |  |  |
|  | Model 3: adjusted for age, education, social class and income. | | | | | | | | | |  |  |  |  |  |  |  |  |  |  |
|  | ^c^ P value for change in difference in excess mortality for those living alone compared to married and cohabiting persons. | | | | | | | | | |  |  |  |  |  |  |  |  |  |  |

| **Table S5.** Relative mortality from selected causes of death (alcohol-related excluded) for living alone vs. married and cohabiting in women aged 15-79 years before (2000-2003) and after (2004-2007) the price reduction. |
| --- |
