# Supplementary material for: Living Alone and Alcohol-Related Mortality: A Population-Based Cohort Study from Finland
Source: PLoS Med. 2011 Sep 20;8(9):e1001094. doi: 10.1371/journal.pmed.1001094 (PMC3176753; doi:10.1371/journal.pmed.1001094)
Supplement: Table S6 — Relative alcohol-related mortality for living alone versus married or cohabiting in men and women aged 15–79 y before (2000–2003) and after (2004–2007) the alcohol price reduction. Overall model in which sex is included as an additional independent variable. (DOC) [file pmed.1001094.s006.doc]

|  | |  |  | Risk ratios for living alone vs. married or cohabiting | | | | | |
| --- | --- | --- | --- | --- | --- | --- | --- | --- | --- |
|  | |  |  | Model 1 | | Model 2 | | Model 3 | |
| Cause of death | | Deaths^a^ | Rate^b^ | RR | 95% CI | RR | 95% CI | RR | 95% CI |
| BEFORE | |  |  |  |  |  |  |  |  |
| Total alcohol-related mortality | |  |  |  |  |  |  |  |  |
| Married or cohabiting | | 3595 | 47.5 | 1.00 |  | 1.00 |  | 1.00 |  |
| Living alone | | 4890 | 219.9 | 4.54 | 4.34-4.74 | 4.30 | 4.11-4.50 | 3.57 | 3.42-3.74 |
| Liver disease | |  |  |  |  |  |  |  |  |
| Married or cohabiting | | 875 | 11.6 | 1.00 |  | 1.00 |  | 1.00 |  |
| Living alone | | 809 | 37.3 | 2.94 | 2.67-3.25 | 2.83 | 2.56-3.13 | 2.39 | 2.16-2.63 |
| Dependence and poisoning | |  |  |  |  |  |  |  |  |
| Married or cohabiting | | 562 | 7.2 | 1.00 |  | 1.00 |  | 1.00 |  |
| Living alone | | 950 | 45.6 | 5.54 | 4.98-6.16 | 5.27 | 4.74-5.87 | 4.25 | 3.82-4.72 |
| Other alcohol-related diseases | |  |  |  |  |  |  |  |  |
| Married or cohabiting | | 196 | 2.6 | 1.00 |  | 1.00 |  | 1.00 |  |
| Living alone | | 356 | 16.6 | 6.24 | 5.22-7.45 | 5.92 | 4.95-7.08 | 4.85 | 4.05-5.80 |
| Cardiovascular diseases | |  |  |  |  |  |  |  |  |
| Married or cohabiting | | 600 | 8.3 | 1.00 |  | 1.00 |  | 1.00 |  |
| Living alone | | 958 | 39.6 | 5.47 | 4.93-6.08 | 5.21 | 4.69-5.80 | 4.47 | 4.02-4.97 |
| Accidents and violence | |  |  |  |  |  |  |  |  |
| Married or cohabiting | | 1097 | 14.3 | 1.00 |  | 1.00 |  | 1.00 |  |
| Living alone | | 1347 | 61.1 | 4.09 | 3.77-4.43 | 3.82 | 3.52-4.15 | 3.26 | 3.00-3.53 |
| AFTER | |  |  |  |  |  |  |  |  |
| Total alcohol-related mortality | |  |  |  |  |  |  |  |  |
| Married or cohabiting | | 3136 | 53.3 | 1.00 |  | 1.00 |  | 1.00 |  |
| Living alone | | 6625 | 269.6 | 4.91 | 4.69-5.15 | 4.63 | 4.42-4.85 | 3.72 | 3.55-3.89 |
| P value^c^ | |  |  |  | 0.008 |  | 0.015 |  | 0.189 |
| Liver disease | |  |  |  |  |  |  |  |  |
| Married or cohabiting | | 862 | 14.5 | 1.00 |  | 1.00 |  | 1.00 |  |
| Living alone | | 1488 | 62.2 | 3.96 | 3.62-4.33 | 3.77 | 3.45-4.13 | 3.04 | 2.78-3.33 |
| P value^c^ | |  |  |  | <0.001 |  | <0.001 |  | <0.001 |
| Dependence and poisoning | |  |  |  |  |  |  |  |  |
| Married or cohabiting | | 494 | 8.3 | 1.00 |  | 1.00 |  | 1.00 |  |
| Living alone | | 1144 | 49.3 | 5.38 | 4.81-6.02 | 5.04 | 4.49-5.64 | 3.87 | 3.45-4.33 |
| P value^c^ | |  |  |  | 0.652 |  | 0.587 |  | 0.266 |
| Other alcohol-related diseases | |  |  |  |  |  |  |  |  |
| Married or cohabiting | | 162 | 2.7 | 1.00 |  | 1.00 |  | 1.00 |  |
| Living alone | | 500 | 21.0 | 7.21 | 5.99-8.68 | 6.80 | 5.63-8.20 | 5.37 | 4.44-6.48 |
| P value^c^ | |  |  |  | 0.245 |  | 0.264 |  | 0.399 |
| Cardiovascular diseases | |  |  |  |  |  |  |  |  |
| Married or cohabiting | | 519 | 9.0 | 1.00 |  | 1.00 |  | 1.00 |  |
| Living alone | | 1299 | 48.5 | 6.07 | 5.44-6.76 | 5.81 | 5.21-6.48 | 4.91 | 4.40-5.48 |
| P value^c^ | |  |  |  | 0.133 |  | 0.151 |  | 0.279 |
| Accidents and violence | |  |  |  |  |  |  |  |  |
| Married or cohabiting | | 872 | 15.1 | 1.00 |  | 1.00 |  | 1.00 |  |
| Living alone | | 1603 | 66.2 | 4.20 | 3.84-4.58 | 3.88 | 3.55-4.24 | 3.17 | 2.90-3.46 |
| P value^c^ | |  |  |  | 0.683 |  | 0.801 |  | 0.801 |
|  | ^a^ Numbers of deaths are those observed in the original sample. | | | | | | | | |
|  | ^b^ Mortality rates (deaths per 100,000) adjusted for age. | | | | | | | | |
|  | Model 1: adjusted for age and sex. | | | | | | | | |
|  | Model 2: adjusted for age, sex, education and social class. | | | | | | | | |
|  | Model 3: adjusted for age, sex, education, social class and income. | | | | | | | | |
|  | ^c^ P value for change in difference in excess mortality for those living alone compared to married and cohabiting persons. | | | | | | | | |

| **Table S6.** Relative alcohol-related mortality for living alone vs. married and cohabiting in men and women aged 15-79 years before (2000-2003) and after (2004-2007) the price reduction. |
| --- |
