# Supplementary material for: Living Alone and Alcohol-Related Mortality: A Population-Based Cohort Study from Finland
Source: PLoS Med. 2011 Sep 20;8(9):e1001094. doi: 10.1371/journal.pmed.1001094 (PMC3176753; doi:10.1371/journal.pmed.1001094)
Supplement: Table S7 — Relative alcohol-related mortality for living alone versus married or cohabiting in men and women aged 15–79 y before (2000–2003) and after (2004–2007) the alcohol price reduction. Adjusted for continuous age term. (DOC) [file pmed.1001094.s007.doc]

|  | |  |  | Risk ratios for living alone vs. married or cohabiting | | | | | | |
| --- | --- | --- | --- | --- | --- | --- | --- | --- | --- | --- |
|  | |  |  | Model 1 | | Model 2 | | Model 3 | | |
|  | | Deaths^a^ | Rate^b^ | RR | 95% CI | RR | 95% CI | RR | | 95% CI |
| MEN, BEFORE | |  |  |  |  |  |  |  | |  |
| Married or cohabiting | | 2804 | 196.2 | 1.00 |  | 1.00 |  | 1.00 | |  |
| Living alone | | 4199 | 664.9 | 5.44 | 5.18-5.72 | 5.18 | 4.93-5.44 | 4.15 | | 3.95-4.37 |
| MEN, AFTER | |  |  |  |  |  |  |  | |  |
| Married or cohabiting | | 2393 | 193.8 | 1.00 |  | 1.00 |  | 1.00 | |  |
| Living alone | | 5571 | 719.6 | 5.86 | 5.57-6.17 | 5.54 | 5.26-5.83 | 4.28 | | 4.06-4.52 |
| P value^c^ | |  |  | 0.051 |  | 0.073 |  | 0.408 | |  |
| WOMEN, BEFORE | |  |  |  |  |  |  |  | |  |
| Married or cohabiting | | 791 | 103.5 | 1.00 |  | 1.00 |  | 1.00 | |  |
| Living alone | | 691 | 184.6 | 2.18 | 1.95-2.43 | 2.11 | 1.89-2.35 | 2.17 | | 1.95-2.42 |
| WOMEN, AFTER | |  |  |  |  |  |  |  | |  |
| Married or cohabiting | | 743 | 102.0 | 1.00 |  | 1.00 |  | 1.00 | |  |
| Living alone | | 1054 | 197.2 | 2.58 | 2.32-2.87 | 2.44 | 2.20-2.71 | 2.48 | | 2.23-2.75 |
| P value^c^ | |  |  | 0.027 |  | 0.062 |  | 0.064 | |  |
|  | ^a^ Numbers of deaths are those observed in the original sample. | | | | | | | |  | |
|  | ^b^ Mortality rates (deaths per 100,000) adjusted for age. | | | | | | | |  | |
|  | Model 1: adjusted for age (continuous). | | | | | | | |  | |
|  | Model 2: adjusted for age (continuous), education and social class. | | | | | | | |  | |
|  | Model 3: adjusted for age (continuous), education, social class and income. | | | | | | | |  | |
|  | ^c^ P value for change in difference in excess mortality for those living alone compared to married and cohabiting persons. | | | | | | | |  | |

| **Table S7.** Relative alcohol-related mortality for living alone vs. married and cohabiting in men aged 15-79 years before (2000-2003) and after (2004-2007) the price reduction. |
| --- |
