# Supplementary material for: Living Alone and Alcohol-Related Mortality: A Population-Based Cohort Study from Finland
Source: PLoS Med. 2011 Sep 20;8(9):e1001094. doi: 10.1371/journal.pmed.1001094 (PMC3176753; doi:10.1371/journal.pmed.1001094)
Supplement: Table S8 — Relative alcohol-related mortality for living alone versus married or cohabiting in men and women aged 15–79 y before (2000–2003) and after (2004–2007) the alcohol price reduction. Adjusted for squared age term. (DOC) [file pmed.1001094.s008.doc]

|  | |  |  | Risk ratios for living alone vs. married or cohabiting | | | | | | |
| --- | --- | --- | --- | --- | --- | --- | --- | --- | --- | --- |
|  | |  |  | Model 1 | | Model 2 | | Model 3 | | |
|  | | Deaths^a^ | Rate^b^ | RR | 95% CI | RR | 95% CI | RR | | 95% CI |
| MEN, BEFORE | |  |  |  |  |  |  |  | |  |
| Married or cohabiting | | 2804 | 196.2 | 1.00 |  | 1.00 |  | 1.00 | |  |
| Living alone | | 4199 | 664.9 | 5.32 | 5.07-5.59 | 5.06 | 4.82-5.32 | 4.06 | | 3.86-4.27 |
| MEN, AFTER | |  |  |  |  |  |  |  | |  |
| Married or cohabiting | | 2393 | 193.8 | 1.00 |  | 1.00 |  | 1.00 | |  |
| Living alone | | 5571 | 719.6 | 5.73 | 5.44-6.03 | 5.41 | 5.14-5.70 | 4.19 | | 3.97-4.42 |
| P value^c^ | |  |  | 0.049 |  | 0.077 |  | 0.421 | |  |
| WOMEN, BEFORE | |  |  |  |  |  |  |  | |  |
| Married or cohabiting | | 791 | 103.5 | 1.00 |  | 1.00 |  | 1.00 | |  |
| Living alone | | 691 | 184.6 | 2.24 | 2.00-2.51 | 2.20 | 1.97-2.46 | 2.26 | | 2.03-2.52 |
| WOMEN, AFTER | |  |  |  |  |  |  |  | |  |
| Married or cohabiting | | 743 | 102.0 | 1.00 |  | 1.00 |  | 1.00 | |  |
| Living alone | | 1054 | 197.2 | 2.67 | 2.40-2.97 | 2.53 | 2.28-2.81 | 2.54 | | 2.29-2.82 |
| P value^c^ | |  |  | 0.018 |  | 0.041 |  | 0.096 | |  |
|  | ^a^ Numbers of deaths are those observed in the original sample. | | | | | | | |  | |
|  | ^b^ Mortality rates (deaths per 100,000) adjusted for age. | | | | | | | |  | |
|  | Model 1: adjusted for squared age. | | | | | | | |  | |
|  | Model 2: adjusted for squared age, education and social class. | | | | | | | |  | |
|  | Model 3: adjusted for squared age, education, social class and income. | | | | | | | |  | |
|  | ^c^ P value for change in difference in excess mortality for those living alone compared to married and cohabiting persons. | | | | | | | |  | |

| **Table S8.** Relative alcohol-related mortality for living alone vs. married and cohabiting in men aged 15-79 years before (2000-2003) and after (2004-2007) the price reduction. |
| --- |
