# Supplementary material for: Living Alone and Alcohol-Related Mortality: A Population-Based Cohort Study from Finland
Source: PLoS Med. 2011 Sep 20;8(9):e1001094. doi: 10.1371/journal.pmed.1001094 (PMC3176753; doi:10.1371/journal.pmed.1001094)
Supplement: Table S9 — Relative alcohol-related mortality for living alone versus married or cohabiting in men and women aged 15–79 y before (2000–2003) and after (2004–2007) the alcohol price reduction. Adjusted for continuous age and squared age term. (DOC) [file pmed.1001094.s009.doc]

|  | |  |  | Risk ratios for living alone vs. married or cohabiting | | | | | | |
| --- | --- | --- | --- | --- | --- | --- | --- | --- | --- | --- |
|  | |  |  | Model 1 | | Model 2 | | Model 3 | | |
|  | | Deaths^a^ | Rate^b^ | RR | 95% CI | RR | 95% CI | RR | | 95% CI |
| MEN, BEFORE | |  |  |  |  |  |  |  | |  |
| Married or cohabiting | | 2804 | 196.2 | 1.00 |  | 1.00 |  | 1.00 | |  |
| Living alone | | 4199 | 664.9 | 5.86 | 5.58-6.16 | 5.51 | 5.25-5.79 | 4.19 | | 3.98-4.41 |
| MEN, AFTER | |  |  |  |  |  |  |  | |  |
| Married or cohabiting | | 2393 | 193.8 | 1.00 |  | 1.00 |  | 1.00 | |  |
| Living alone | | 5571 | 719.6 | 6.19 | 5.88-6.52 | 5.77 | 5.48-6.08 | 4.21 | | 3.99-4.44 |
| P value^c^ | |  |  | 0.162 |  | 0.208 |  | 0.925 | |  |
| WOMEN, BEFORE | |  |  |  |  |  |  |  | |  |
| Married or cohabiting | | 791 | 103.5 | 1.00 |  | 1.00 |  | 1.00 | |  |
| Living alone | | 691 | 184.6 | 2.77 | 2.48-3.10 | 2.79 | 2.49-3.12 | 3.01 | | 2.69-3.36 |
| WOMEN, AFTER | |  |  |  |  |  |  |  | |  |
| Married or cohabiting | | 743 | 102.0 | 1.00 |  | 1.00 |  | 1.00 | |  |
| Living alone | | 1054 | 197.2 | 3.19 | 2.86-3.55 | 3.16 | 2.84-3.52 | 3.25 | | 2.93-3.60 |
| P value^c^ | |  |  | 0.027 |  | 0.062 |  | 0.260 | |  |
|  | ^a^ Numbers of deaths are those observed in the original sample. | | | | | | | |  | |
|  | ^b^ Mortality rates (deaths per 100,000) adjusted for age. | | | | | | | |  | |
|  | Model 1: adjusted for age (continuous) and squared age. | | | | | | | |  | |
|  | Model 2: adjusted for age (continuous) and squared age, education and social class. | | | | | | | |  | |
|  | Model 3: adjusted for age (continuous) and squared age, education, social class and income. | | | | | | | |  | |
|  | ^c^ P value for change in difference in excess mortality for those living alone compared to married and cohabiting persons. | | | | | | | |  | |

| **Table S9.** Relative alcohol-related mortality for living alone vs. married and cohabiting in men aged 15-79 years before (2000-2003) and after (2004-2007) the price reduction. |
| --- |
|
